# Efficacy of the ‘hole-punch’ technique in recurrent auricular hematoma after excisional hemangioma: a case report

**DOI:** 10.1093/jscr/rjaf124

**Published:** 2025-03-09

**Authors:** Ali S Al Shahrani, Abdulrazaq M Alshammari, Nader S Alharbi, Mohammed Hazazi, Eman Almashharawi

**Affiliations:** Department of Otorhinolaryngology Head and Neck Surgery, Armed Forces Hospital, Riyadh, Saudi Arabia; Department of Otorhinolayrngology Head and Neck Surgery, King Salman Hospital, Riyadh, Saudi Arabia; Department of Otorhinolayrngology Head and Neck Surgery, Shaqra University, Riyadh, Saudi Arabia; Department of Otorhinolaryngology Head and Neck Surgery, Armed Forces Hospital, Riyadh, Saudi Arabia; Department of Otorhinolaryngology Head and Neck Surgery, Armed Forces Hospital, Riyadh, Saudi Arabia

**Keywords:** ‘hole-punch’ technique, auricular hematoma, wound healing, recurrent

## Abstract

Auricular hematoma commonly arises from trauma, often leading to significant morbidity if not addressed promptly. Traditional management techniques frequently result in high recurrence rates and may not prevent the development of cauliflower ear. We describe a case of a 12-year-old girl with autism and attention-deficit/hyperactivity disorder, presenting with a persistent, firm, and dark red mass on her right pinna following previous treatment for an auricular hemangioma. The innovative ‘hole-punch’ Technique was employed to manage this recurrent auricular hematoma. This technique, utilizing a dermal punch tool to create strategic excision sites, facilitated effective hematoma evacuation and promoted fibrosis. The procedure yielded excellent aesthetic results without complications, and no recurrence was noted at follow-up. The ‘hole-punch’ technique offers a promising alternative for managing recurrent auricular hematomas in pediatric patients, potentially reducing recurrence rates and improving cosmetic outcomes. Further studies are required to validate these preliminary findings.

## Introduction

Auricular hematoma, commonly resulting from trauma, poses a risk of significant morbidity if not promptly and effectively managed [1]. The mechanism of injury is most often a blunt shearing force that disrupts the tightly adherent perichondrium (and therefore blood supply) to the underlying cartilage or iotrogenic during surgeries as in the case presented [2]. This results in accumulation of blood in the subperichondrial space [3, 4]. Traditional approaches, such as aspiration or incision and drainage, accompanied by pressure dressing, are fraught with high recurrence rates and do not consistently prevent cauliflower ear—a deformity characterized by fibrocartilage overgrowth post-injury [1, 2].

Herein, we delineate the utilization of the ‘hole-punch’ technique in treating a 12-year-old girl presenting with a recurrent auricular hematoma, underscoring the procedure’s success and the favorable postoperative outcomes. This case contributes to the evolving evidence base advocating for innovative ENT solutions and holds the potential to shift the paradigm of auricular hematoma management in the pediatric demographic.

## Case presentation

**Figure 1:**
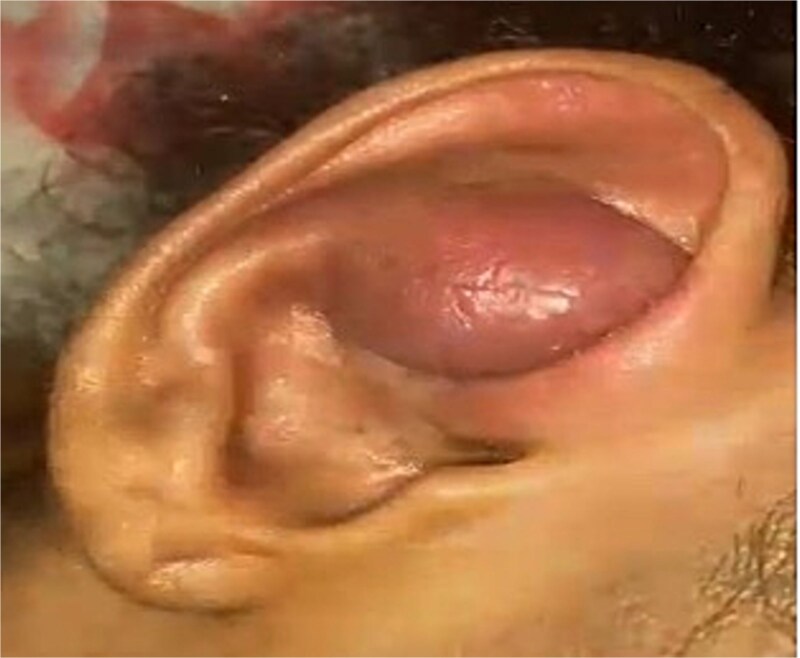
This figure showed a firm, progressively darkening mass is observed on the right ear pre-operation.

A 12-year-old girl reported with a right auricular lump that had been present since she was two years old. It was classified as a hemangioma (Fig. 1). The mass was firm, non-mobile, adhered to the skin, and remained the same size while bending forward. The examination of the left ear was normal.

**Figure 2:**
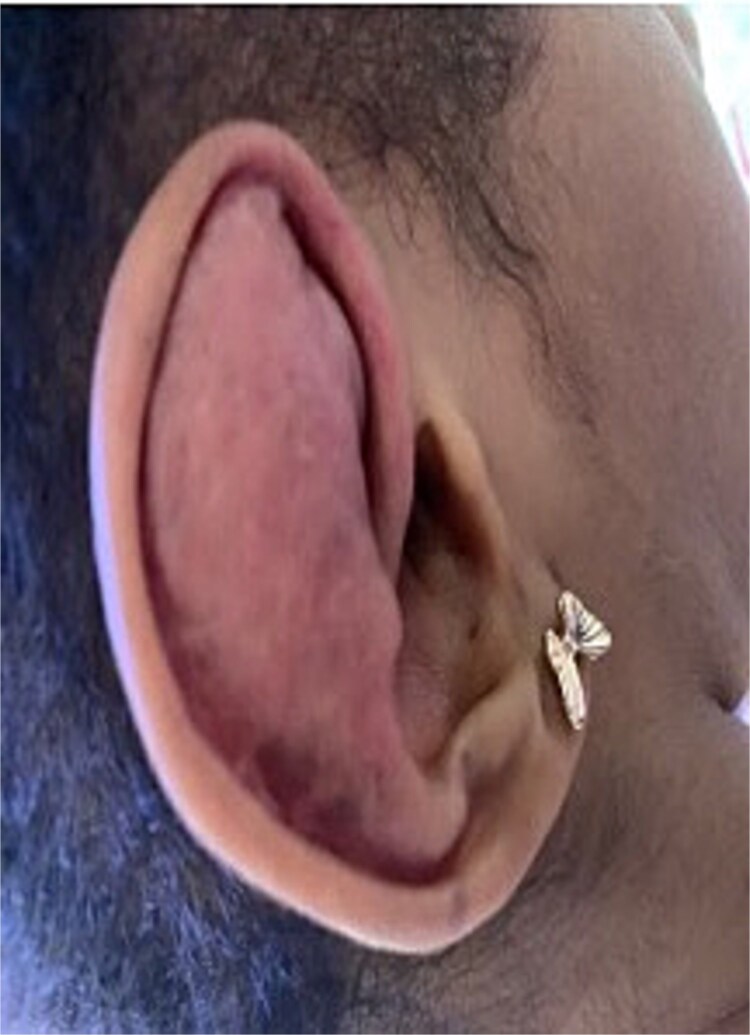
Shows a persistent, enlarging right auricular mass consistent with a recurrent hematoma following prior surgical excision.

The patient underwent right auricular lump and some of the associated cartilage removed. Following the procedure, she developed a hematoma at the surgical site, which was originally aspirated and treated conservatively. However, the hematoma reappeared, and the mass gradually expanded in size (Fig. 2).

**Figure 3:**
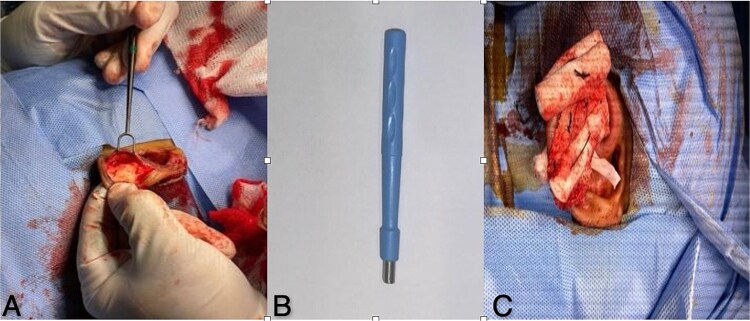
(A) Surgical approach incision at scapha-helical rim exposes split cartilage and hematoma. (B) Dermal punch tool specialized instrument used for hole punch technique to remove affected cartilage. (C) Post-operative care suture, drain, cotton-wool roll, and vaseline gauze maintain ear shape during recovery.

Given the recurrence, the patient underwent a procedure using the ‘hole-punch’ technique. An incision was done at the scapha-helical rim junction to identify the split cartilage impacted by the hematoma (Fig. 3A). The front layer of the deformed cartilage was removed, and a 3 mm dermal punch was used to make holes in the remaining cartilage layer (Fig. 3B). A penrose drain was inserted, and a dental cotton-wool roll was used for 13 days (Fig. 3C). Antibiotics prophylaxis was given for 2 weeks, The patient was Followed up after 3 days with no sign of infection and drain was removed.

**Figure 4:**
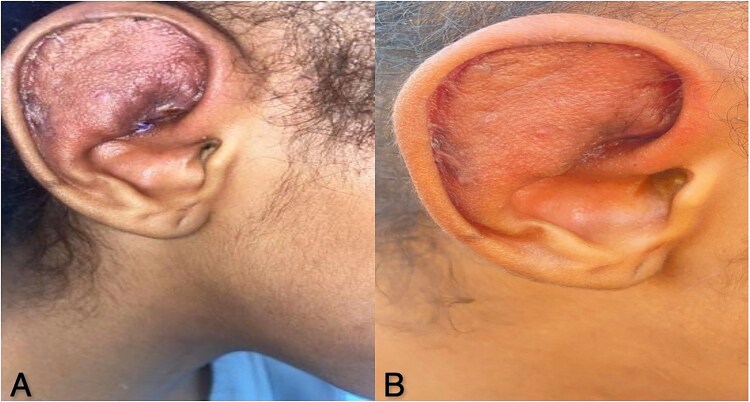
(A) Excellent post-operative result, the surgical site is well-integrated, indicating the effectiveness of the hole-punch technique. (B) Favorable outcome at 2 months, the favorable outcome is maintained 2 months after the procedure.

The outcome of the procedure was highly favorable, indicating the potential efficacy of this technique in managing similar cases (Fig. 4).

## Discussion

Auricular hematomas in young people patients provide unique therapeutic problems, notably in terms of recurrence and the development of cauliflower ear [1, 2]. While basic techniques like needle aspiration are often used, they frequently result in high recurrence rates in such patients [5]. More successful therapies for recurring or massive hematomas include incision, drainage, and debridement, which is often combined with compression approaches [5].

In this example, the patient first had needle aspiration, but the hematoma resurfaced. Subsequent attempts at aspiration, incision, drainage, and debridement failed to prevent recurrence. Only using the ‘hole-punch’ method resulted in a considerable improvement.

The ‘hole-punch’ approach has significant advantages over previous procedures. The use of a dermal punch device enables for precise tissue removal at the surgery site while resulting in minimal harm to adjacent tissues, lowering the risk of infection and scarring—an important factor in pediatric patients whose cosmetic outcomes are highly valued [1]. Furthermore, the technique’s ability to promote fibrosis and facilitate the attachment of the auricular perichondrium to the underlying cartilage reduces the chance of fluid re-accumulation.

In this case, the ‘hole-punch’ approach was effectively employed to treat the patient’s recurrent auricular hematoma, with a positive outcome defined by the absence of recurrence, maintenance of normal ear contour, and absence of sequelae such as infection or severe scarring. The patient also showed a high level of postoperative tolerance, which is noteworthy given her cognitive and developmental problems.

While this single case study indicates the potential benefits of the ‘hole-punch’ strategy, wider usage of the treatment would demand more extensive data to confirm its efficacy and safety. Randomized controlled trials comparing this strategy to alternative management procedures, as well as rigorous long-term follow-up research, are needed to determine the method’s long-term effectiveness.

## Conclusion

The successful application of the ‘hole-punch’ technique in this case suggests that it may offer a superior alternative to traditional methods for treating recurrent auricular hematomas in pediatric patients. The technique’s potential for reducing recurrence rates, minimizing cosmetic deformity, and improving patient tolerance makes it a compelling option in pediatric ENT management. Moving forward, further investigation will be essential to validate these findings and incorporate the ‘hole-punch’ technique into standard practice for auricular hematoma management.

## Data Availability

The data used to support the findings of this study are available upon request.

## References

[1] Lamb MM, Mims MM, Clark JM. “Hole-punch” technique for recurrent auricular hematomas. Laryngoscope 2023;133:814–7. 10.1002/lary.30298. 35861159

[2] Greywoode JD, Pribitkin EA, Krein H. Management of auricular hematoma and the cauliflower ear. Facial Plast Surg 2010;26:451–5. 10.1055/s-0030-1267719. 21086231

[3] Lee D, Sperling N. Initial management of auricular trauma. Am Fam Physician 1996;53:2339–44. Erratum in: Am Fam Physician 1996;54:468. 8638510

[4] Dalal PJ, Purkey MR, Price CPE, et al. Risk factors for auricular hematoma and recurrence after drainage. Laryngoscope 2020;130:628–31. 10.1002/lary.28310. 31621925

[5] Giles WC, Iverson KC, King JD, et al. Incision and drainage followed by mattress suture repair of auricular hematoma. Laryngoscope 2007;117:2097–9. 10.1097/MLG.0b013e318145386c. 17921905

